# Influence of transient spatial attention on the P3 component and perception of painful and non-painful electric stimuli in crossed and uncrossed hands positions

**DOI:** 10.1371/journal.pone.0182616

**Published:** 2017-09-05

**Authors:** Karolina Świder, Eligiusz Wronka, Joukje M. Oosterman, Clementina M. van Rijn, Marijtje L. A. Jongsma

**Affiliations:** 1 Institute of Psychology, Jagiellonian University, Kraków, Poland; 2 Donders Institute for Brain, Cognition & Behaviour, Radboud University, Nijmegen, The Netherlands; 3 Behavioural Science Institute, Radboud University, Nijmegen, The Netherlands; Centre de neuroscience cognitive, FRANCE

## Abstract

Recent reports show that focusing attention on the location where pain is expected can enhance its perception. Moreover, crossing the hands over the body’s midline is known to impair the ability to localise stimuli and decrease tactile and pain sensations in healthy participants. The present study investigated the role of transient spatial attention on the perception of painful and non-painful electrical stimuli in conditions in which a match or a mismatch was induced between skin-based and external frames of reference (uncrossed and crossed hands positions, respectively). We measured the subjective experience (Numerical Rating Scale scores) and the electrophysiological response elicited by brief electric stimuli by analysing the P3 component of Event-Related Potentials (ERPs). Twenty-two participants underwent eight painful and eight non-painful stimulus blocks. The electrical stimuli were applied to either the left or the right hand, held in either a crossed or uncrossed position. Each stimulus was preceded by a direction cue (leftward or rightward arrow). In 80% of the trials, the arrow correctly pointed to the spatial regions where the stimulus would appear (congruent cueing). Our results indicated that congruent cues resulted in increased pain NRS scores compared to incongruent ones. For non-painful stimuli such an effect was observed only in the uncrossed hands position. For both non-painful and painful stimuli the P3 peak amplitudes were higher and occurred later for incongruently cued stimuli compared to congruent ones. However, we found that crossing the hands substantially reduced the cueing effect of the P3 peak amplitudes elicited by painful stimuli. Taken together, our results showed a strong influence of transient attention manipulations on the NRS ratings and on the brain activity. Our results also suggest that hand position may modulate the strength of the cueing effect, although differences between painful and non-painful stimuli exist.

## Introduction

Attention is a cognitive function crucial for the selection of sensory events that consequently enter our awareness. Spatial attention is the ability to selectively process stimuli at a specific location. To determine the location of external stimuli, the brain has to be able to represent space according to different frames of reference. The internal (skin-based), and external reference frames are two specific types of space-representations [1–3]. The skin-based reference frame is associated with the position of the receptive fields of the body and is reflected in the spatial arrangement of neurons in the primary and secondary somatosensory cortex [4]. The external reference frame is related to the estimation of our body’s posture in relation to its external surroundings, based mostly on visual information [2,5]. Moreover, proper localisation of pain and tactile stimuli is determined by integrating information from different modalities and constructing spatial representations of the body parts and the surrounding space [5–7].

The internal and the external frames of reference can be brought into conflict by crossing one’s arms over the body’s midline. When we cross our hands, the left hand (reflecting the skin-based reference frame) is located in the right space (reflecting the external reference frame), inducing a conflict between the skin-based and the external reference frames [3,8]. The resulting conflict was shown to impair tactile [4,5,8–10] and also painful [11] stimulus localisation. For example, in studies where participants performed judgements about the order of the stimuli presented in short temporal succession to both hands (the temporal order judgment (TOJ) task), crossing the hands decreased the ability to determine the stimuli order (crossed-hand deficit) [5,12–14]. Localisation of the tactile stimuli in crossed hands posture was impaired due to difficulties in integrating conflicting information from different spatial reference frames [4,14]. Moreover, previous studies have also shown that a conflict between both frames of reference reduces pain in healthy participants (crossed-hands analgesia) [15–17].

Apart from behavioural ratings and judgements, extracting event-related potentials (ERPs) in response to painful and non-painful electric stimuli from the on-going electroencephalogram (EEG) provides an excellent means to study directly the neural responses associated with either congruent or incongruent internal and external reference frames [18–20]. For example, Gallace et al. [15] (2011) reported that during the crossed hands condition, a decreased peak amplitude of the N2-P2 ERP component was observed together with decreased subjective ratings of both laser-evoked painful and electrically evoked non-painful stimuli (crossed-hands analgesia). However, the magnitude of early components of the response elicited by somatosensory stimuli (e.g., the N1 wave of laser-evoked) was not affected by crossing the hands. The authors assumed that the obtained behavioural (crossed-hands analgesia) and neural effects (decreased ERP P2-N2) were associated with disrupted multimodal cortical processing of somatosensory information caused by mismatch of skin-based and external reference frame.

One way to modulate participants’ spatial attention is to use cues preceding the presentation of the stimuli of interest. A classic paradigm for the study of spatial attention was originally developed by Posner [21]. In addition to many studies of visual perception, pain perception has been shown to be modulated by manipulating spatial attention using the Posner paradigm. Ryckeghem and colleagues [22], who presented visual stimuli on either the same side or the opposite side of painful stimuli, found that participants rated their sensations as more painful when the visual cue was presented on the same side as the painful stimulus. Moreover, other studies investigating the influence of spatial attention manipulations on pain perception have reported that focusing attention on painful stimuli exaggerated the sensation of pain [6,23], and focusing attention away from the stimulus location (either on another task or on another perceptual object) reduced pain and resulted in increased response latencies [24,25].

Apart from modulating the subjectively reported experience of pain, attention affects ERPs elicited by painful stimuli. In general, pain ERPs are characterised by a negative peak around 130–240 ms poststimulus and a positive peak around 230–390 ms poststimulus [26]. Crucially, brain responses to incoming electrical stimuli appear in the time range in which cognitive potentials, such as the P300 (P3), are usually observed [27–31]. Desmedt et al. [32] first described that relevance and occurrence probability of somatosensory stimuli are associated with P3 (P300) elicitation in human EEG brain. The P3 component is a family of waves that peaks at around 250–400 ms and is intimately related to task performance [31,33]. There are at least two variants of this component—P3a and P3b [24,26,29,30,34–37]. The amplitude of the P3a component is usually smaller than P3b’s amplitude, with shorter latency and more frontal topography (larger in Cz than Pz). What is important in the context of this study is that the P3a component is thought to be an index of involuntary attention allocation to novel, salient and threatening stimuli that appear outside of the attention focus [33–36,38], and the latency of the P3 component is thought to be an index of stimulus processing speed [39]. As was shown in studies by Legrain et al. [24,27], the laser-evoked P2 component (probably a P3a component; for discussion see [40–42]) was enhanced for rare stimuli as compared to frequent stimuli. Other studies have reported similar results, indicating that standard stimuli delivered to the unattended location elicited ERPs of greater amplitude compared to ERPs elicited by standard stimuli delivered to the attended location [41–43]. Accordingly, each electrical stimulus in the present study was preceded by a visual cue (leftward and rightward arrows) that indicated the spatial location of the stimulus application (right or left side; congruent cueing—CC). In addition, to check the involvement of attentional processes, incorrect cues were occasionally presented (i.e. 20% of the trials), which preceded an electrical stimulus that was delivered to the opposite spatial location than was indicated by the cue (right cue—left location or left cue—right location; incongruent cueing—IC). Thus, in the present study, we expected a modulation of both the peak amplitudes and peak latencies of the P3 component elicited by painful and non-painful electric stimuli when attention was manipulated by cues [24,30,34–37,44].

The use of cues makes the present study different from the study of Gallace et al. [15], where participants’ hands were stimulated in an unpredictable manner without any additional attentional focus on the side of laser painful and electric non-painful stimulus application. In other words, the spatial location (right or left side) of the subsequent stimulus was unknown to the participants. To our knowledge, there are no data indicating whether crossed-hands analgesia can be also elicited when the participants’ attention is directed to the right or left spatial location where electrical stimuli (pain or non-painful) are expected to be delivered. Moreover, the design of our study required participants to switch their attention from trial to trial and the occasional incorrectly cued stimulus induced a re-allocation of attention to a different location in space. Consequently, such a design allowed us to explore the effects of transient spatial attention [44]. Other studies that report the effects of the processing of tactile stimuli (with either congruent or incongruent internal and external reference frames) in the time ranges of 80–160 and 200–300 ms post stimuli usually use a sustained manipulation of spatial attention [18–20]. In these studies, by contrast to ours, participants’ attention is usually maintained on one specific limb for the entire experimental block, and stimuli are consistently delivered to either the attended or unattended side (a sustained manipulation of spatial attention) [18–20]. Thus it is worth studying whether the transient attentional effect is modulated by crossing the hands.

We focused our analysis on P3 components (probably P3a) due to the fact that the magnitude of late components (the N2-P2 wave of laser-evoked potentials), contrary to early ones (the N1 component), was shown to be affected by crossing the hands [15]. Moreover, P3a is one of three components (next to contralateral temporal negativity—CTN and fronto-central negativity—FCN) that is elicited by electrical painful stimuli that appear to be involved in detecting and orienting attention towards unattended somatic threats [36,38]. The relation between the P3 component and attention allocation has been well documented [33–36,38,45] but the functional role of this relation is still under investigation.

To sum up, the present study’s aim was to investigate the effect of transient spatial attention on subjective intensity ratings of pain and tactile stimuli and on the P3 component elicited by both types of electrical stimuli. Pursuing this aim, we used congruent and incongruent cues. Additionally, the second goal of this study was to compare these effects under conditions of matched and mismatched internal and external frames of reference (i.e. a crossed and an uncrossed hands condition). Consequently, the current experiment created four different conditions for each stimulus intensity (uncrossed congruent/incongruent cue conditions and crossed congruent/incongruent cue conditions). Based on previous studies, we expected that, for both painful and non-painful stimuli (S1 Fig):

1. The NRS ratings will be higher in the condition where the stimulus location is correctly signalled (congruent cueing condition) as compared to the condition where the stimulus location is incorrectly signalled (incongruent cueing condition).
2. In the crossed hands condition, NRS ratings will be lower compared to the uncrossed hands condition (i.e. crossed-hands analgesia).
3. The highest NRS pain ratings will occur after congruent cues compared to incongruent cues specifically in the uncrossed hands condition, because in this condition there is a match between the internal reference frame and the external reference frame. In the crossed hands condition the cueing effect should be substantially decreased due to an impaired ability to localise stimuli caused by a mismatch between both frames of reference.
4. The ERP P3 component will have a higher amplitudes and will be delayed in latencies after incongruently cued stimuli compared to congruently cued stimuli, because of the re-allocation of transient spatial attention.
5. The ERP P3 component amplitudes will be decreased by crossing the hands, due to a mismatch between the internal and external frame of reference.
6. The ERP P3 amplitudes elicited by stimuli preceded by incongruent cues will be higher in uncrossed conditions compared to the crossed conditions.

## Material and methods

### 2.1. Participants

Twenty-five right-handed volunteers (4 males and 21 females) aged between 19 and 29 (mean = 22.8 ± 2.8 years), took part in the experiment. The sample size was based on a literature review of EEG and pain research–the number of participants that usually volunteer is between twenty and twenty-five [46–49] and between fifteen and twenty [11,44,50] or even twelve [15,51–53]. The volunteers were recruited amongst the student population of the Radboud University Nijmegen, the Netherlands. All of the participants were healthy, free of pain, and not taking any medication. Three female participants were excluded from the analyses due to excessive muscle and/or eye artefacts in the EEG. The data from the remaining 22 participants were further analysed (mean age = 23.1 ± 2.9). All participants provided written informed consent prior to the experiment. The participants were informed that they were participating in a study of low and high electrical stimulus perception in two hand positions–crossed and uncrossed. They were also informed that they could stop participating at any point during the study without giving a reason. The participants received remuneration for their participation in the study. The study was approved by the Ethics Committee Faculty of Social Sciences of Radboud University in Nijmegen (ECG2012-1301-005) and by the Research Ethics Committee at the Institute of Psychology of Jagiellonian University.

### 2.2. Procedure

The experiment was performed in a sound-attenuated laboratory room where the participants were seated in a comfortable armchair in front of a computer screen (60 Hz refresh rate) at a distance of roughly 50 cm, with both of their hands resting on the desk. The study consisted of 2 phases: a preparation phase and a testing phase, lasting around 35 and 55 minutes, respectively.

#### 2.2.1. Painful and non-painful electrical stimuli

The magnitudes of the stimuli (painful and non-painful) were individually determined for each of the participants during the preparation phase. Two intensities of electrical stimuli were used in the experiment, painful and non-painful electric shocks, which were delivered by two Constant Current High Voltage Stimulators (200 μs duration; Digitimer, Welwyn Garden City, England, Model DS7AH and DS7A) through two concentric surface electrodes for electrical stimulation of nociceptive nerves (K² stimulating electrodes; Inomed, Germany). Each of the electrodes was attached to the outer side of the right or left hand between the thumb and the index finger, over the superficial branch of the radial nerve. During the preparation phase, the participants received a series of electrical stimuli of increasing and decreasing intensity, delivered to the nondominant hand. The participants’ task was to rate the magnitude of the stimuli verbally on a modified Numerical Rating Scale (NRS). The anchors used in the scale were 0 –*no electrical sensation*; 1, the sensation threshold–*‘I start to feel something’*; 4, the pain threshold–‘*It starts to be painful’*; and 10, the maximum pain tolerance–‘*the strongest painful sensation imaginable’*. To the participants, the NRS score of 10 was explained as a painful stimulus that he or she does not want to receive anymore. The scale resembles the one used in the study of Romero et al [54]. On this scale, values of 1, 4, and 10 indicate the somatosensory threshold (detection threshold—DT), the pain threshold (PT) and the pain tolerance threshold (PTT), respectively. We defined three NRS ratings for non-painful stimulation (from 1–3 on NRS) and used 4 on NRS to determine the pain threshold (PT). We were interested in ratings representing non-painful (3 on NRS) up to highly painful but tolerable stimuli (rating 8 on NRS).

Stimuli delivered in the preparation phase started at 0.1mA. Then the current was increased by 0.1mA per step to determine the DT. Subsequently, the current was decreased until the participant was unable to detect the stimulus. The cycle of increasing and decreasing the stimulus was repeated until a stable threshold was obtained. This required only on average 2–3 increase/decrease cycles for most of the participants. After the DT was determined, the current was subsequently increased in increments of 0.2 mA until the PT was reached, and subsequently decreased to the DT. This step was repeated three times before taking the average of the two scores for the second and third repetition of a ‘3’ NRS rating of a non-painful stimulus. Next, the current was increased in increments of 0.5 mA to detect the pain tolerance threshold (an NRS score of 10) and decreased to the PT. This step was repeated three times before taking the average of the two scores for the second and third repetition of an ‘8’ NRS rating as a painful stimulus. In doing so, the intensities of electrical stimuli that were individually selected for each participant varied: the means for painful and non-painful stimuli intensities were, respectively: 12.54 ± 2.12 mA and 3.99 ± 1.12 mA; *t*(24) = -17.78, *p* < .001). Stimuli at intensities identified as non-painful during the calibration phase were never reported as painful during the experiment.

#### 2.2.2. Electroencephalographic recordings and EEG preprocessing

Electroencephalography (EEG) was recorded from 64 Ag/AgCl electrodes (Acticap, Brain Products, Munich, Germany) according to the international 10–20 system. The reference electrode was located on the right mastoid. Four electrooculographic electrodes (EOGs) were attached to the external canthi of both eyes and above and below the left eye to monitor horizontal and vertical eye movements. The EOG ground electrode was placed on the nose.

The impedance of the EEG electrodes was kept below 20 kΩ. In total, four hundred EEG trials were presented in the two conditions (non-painful and painful electrical stimuli) for each participant. Signals were recorded with a Brain Vision Recorder (Brain Products) using a 150 Hz low-pass filter, with a time constant of 10 s (0,016 Hz) and a 500 Hz sampling frequency. The EEG data were re-referenced off-line to linked mastoids. Subsequently, the EEG signal was filtered (bandpass 0.016–45 Hz; 24 dB) and epoched into 700 ms intervals (epochs between 200 ms before and 500 ms after stimulus onset). All epochs were corrected for eye-movements using a method developed by Gratton et al. [55]. Next, trials with EEG activity exceeding ±100 μV were semi-automatically rejected. The number of rejected trials remained low (mean = 0.87; SD = 1.77). Artefact-free single trials were averaged per participant and with respect to the stimulus delivery condition. All EEG preprocessing was conducted using the Brain Vision Analyzer (Brain Products).

The P3 peak amplitude component was defined as the most positive deflection within the time window, 250–400 ms after stimulus onset respecting the guidelines of Picton et al. [56]. Only data from midline sites were further analysed (Fz, FCz, Cz, CPz, Pz) based on the literature [33,35,39,57–59] and by visual inspection in order to contrast late components of pain- and non-pain-related somatosensory processing. The P3 latency was determined as the time point of maximum positive amplitude on the Cz electrode in the 250–400 ms window. We analysed P3 peak amplitudes recorded from five vertex electrodes in order to compare the magnitude of P3 peak amplitude between electrodes and to be able to indicate if the P3a component was elicited. In contrast to the P3b component, the P3a component is larger at the Cz electrode than at the Pz electrode.

#### 2.2.3. Testing phase

At the beginning of the testing phase, the participants were asked to place both hands on the table at a distance of 40 cm from each other. After that, the stimulation electrodes were attached to their right and left hands. Next, a large wooden tabletop (59×50×15 cm) was slid into place above the arms of the participants to block them from view. The participants were verbally instructed how properly to keep their arms in the uncrossed or crossed positions, before the start of the experiment and between all blocks during the testing phase. The white line in the middle of the wooden screen served as the reference point to separate the right and left side. The participants were left alone in the laboratory room for the remainder of the testing phase but were observed through a camera and received instructions between blocks via a speaker.

The testing phase involved a total of 400 trials (200 trials for each stimulus type) presented in 16 blocks. There were separate blocks for each stimulus intensity: eight blocks with painful stimuli and eight blocks with non-painful stimuli. In 50% of the blocks the participants were instructed to keep their hands uncrossed, and for the other 50% they were asked to cross their hands. Thus, four blocks of painful stimuli were delivered to crossed hands and another four blocks of painful stimuli were delivered to uncrossed hands. Similarly, four blocks of non-painful stimuli were delivered to crossed hands and four blocks of non-painful stimuli to uncrossed hands. The order of the blocks was counterbalanced between the participants. Within a block only one hand position was required, and no more than two consecutive blocks were used with the same hand position.

Half of the stimuli were delivered to the left hand and half to the right hand. The maximum number of consecutive stimuli delivered to one hand was three. Intertrial intervals (ITI) lasted on average 7.00 ± 1.65 s (mean ± SD). Such intervals are considered to be large enough to observe reproducible ERPs.

**Fig 1:**
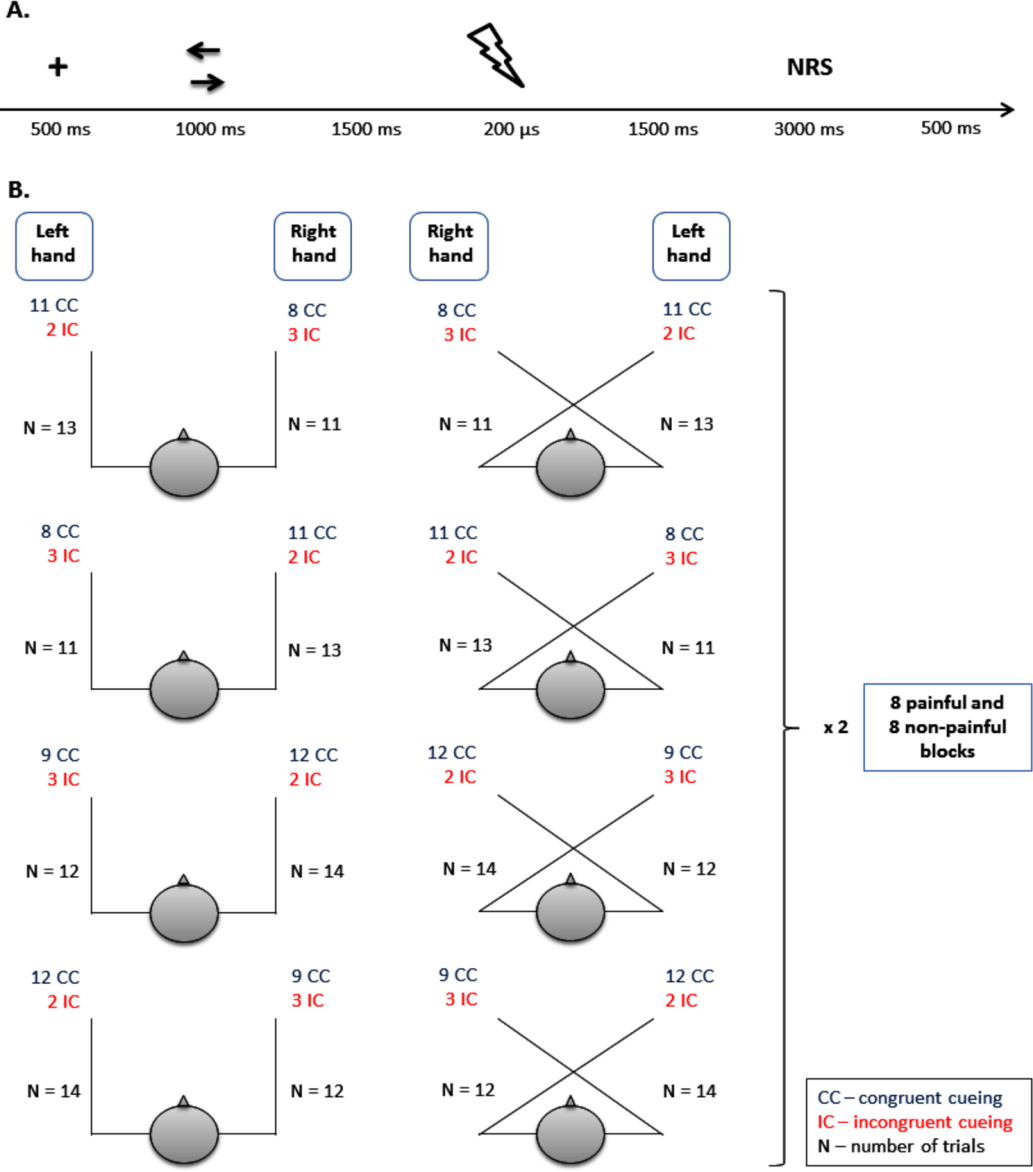
**Overview of trial timing (A) and congruent and incongruent cue schemas used in the experiment.** Ad A. The “+” represents the fixation cross; the right and left arrows represent cue stimuli (during the one trial one of the arrows was presented); the lightning flash represents the electric shock. The length of the NRS epoch was about 3 seconds, which included the time for the participant to evaluate the stimulus plus time for the experimenter to record the response. Ad B. Eight blocks consisted of 19 congruent and 5 incongruent cue trials and the remaining eight blocks consisted of 21 congruent and 5 incongruent cue trials. The same schema was used for the painful and non-painful stimulus blocks. In total, there were 16 blocks (8 painful and 8 non-painful stimulus blocks).

Both types of electrical stimuli were preceded by a sequence of two visual stimuli presented at the centre of a computer screen. These stimuli were a white fixation cross (32×32 pixels) and white arrows pointing to the right or the left (both 32×32 pixels), presented on a black background for 500 ms and 1000 ms, respectively. See Fig 1A for an overview of the trial timing.

**Fig 2:**
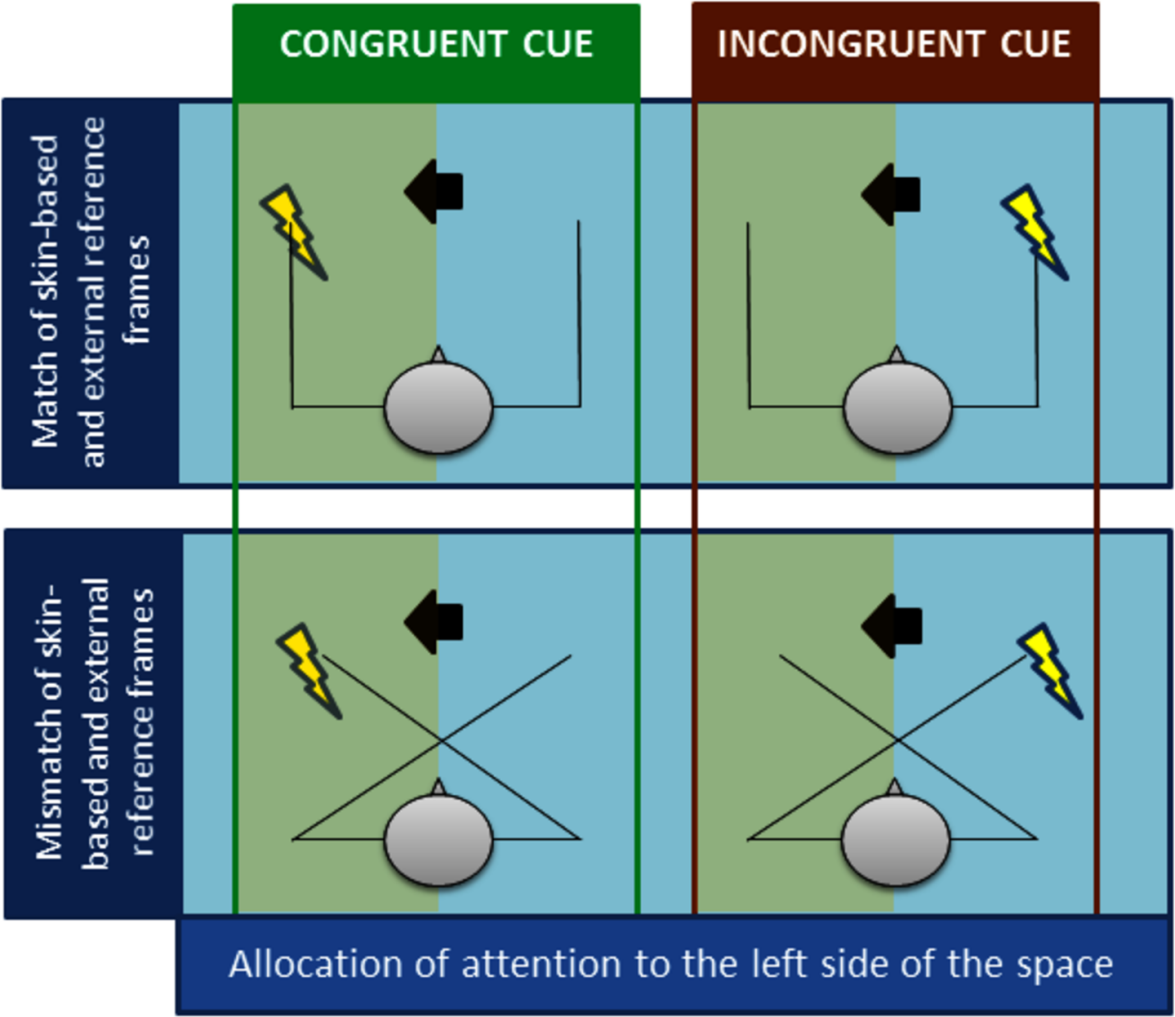
The four conditions of the experiment. In the uncrossed hands condition (top panel) we observe a match between skin-based and external frames of reference (the left hand is placed in the left space). In crossed hands condition (bottom panel) the left hand (reflecting the skin-based reference frame) is located in the right space (reflecting the external reference frame), inducing a conflict between both reference frames. Congruent cueing (left green panel): Left cue allocates participants attention to the left spatial location where stimulus was predicted to appear. Incongruent cueing (right red panel): Left cue allocates participants attention to the left spatial location where stimulus was predicted to appear but it was applied to the right hand. Note that during the experimental phase vision of the hands was prevented and two types of cues were used (leftward and rightward arrows); left arrow represents cue; the lightning flash represents the electric shock; green shade represents the side of allocation of attention.

The presentation of the arrow directed the participants’ attention to the spatial location of subsequently presented electrical stimuli (right or left side) (Fig 2).

In 80% of the trials, the cue correctly indicated the upcoming side of space of the stimulation (congruent cueing—CC), and in 20% of trials the cue incorrectly indicated where the stimulus would be delivered (incongruent cueing—IC). A specific schema of the number of subsequent congruent and incongruent cues was used (for more details, see Fig 1B). The congruent and incongruent cue schemas were presented in a pseudo-random order in each of the 16 blocks of the experiment. The stimuli presented in incongruent cue trials were always preceded by at least one congruent cue and were never presented in the first positions within a block. Each block consisted of a different number of trials in order to avoid habituation to the length of the block and to avoid expectancy reactions with respect to the last couple of trials and thus avoiding confounding ERP effects (for more details, see Fig 1B). The timing of events preceding stimulation was not variable in order to avoid the situation where electrical stimuli would be delivered in an unpredictable manner. Otherwise, it would have had a huge impact on ERP components and pain ratings [60,61].

The participants were instructed at the beginning of each recording block: (1) to pay attention to the information about the stimulation intensity and their hands’ position; (2) to maintain their gaze at the fixation point; and (3) to pay attention to the arrow stimulus that would indicate to which side the electrical stimulus would be delivered. In order to examine if the crossed hands position significantly reduced the perceived intensity of the painful stimuli, the participants rated the intensity of each of the painful and non-painful stimuli using a verbal NRS. Moreover, the participants were instructed not to move their head or eyes, and to blink as little as possible, especially during stimuli presentation.

### 2.3. Statistical analysis

We analysed the data obtained from painful and non-painful trials separately due to the fact that both the NRS ratings and the ERP P3 component amplitudes elicited by painful stimuli are known to be larger than the NRS ratings and the P3 amplitudes elicited by non-painful stimuli [29].

For behavioural data, to address the first and second aim of the study and to verify hypotheses 1–3, we performed two separate General Linear Model (GLM) repeated measures analyses: first for painful NRS ratings and second for non-painful NRS ratings, using Cue (2 levels: congruent and incongruent) and Hand Position (2 levels: crossed and uncrossed) as within-subject factors. The statistically significant main effect of Cue and Hand Position tested our hypotheses 1 and 2, respectively. In the case where statistically significant interaction of factors Cue and Hand Position was observed, the *F* tests were followed by planned comparisons (appropriate planned contrasts by means of F tests) to test our a-priori defined third hypothesis. We investigated whether a cueing effect was present in both hand positions by performing planned comparisons for congruent versus incongruent cues separately in the uncrossed and crossed hands condition.

Statistical analyses of the EEG data were restricted to the EEG signals recorded from the midline electrodes Fz, FCz, Cz, CPz and Pz due to the fact that amplitudes of the P3 component are typically highest over the midline electrodes [33,35,39,57,58]. To address the first and second aim of the study and to verify hypotheses 4–6, the P3 peak amplitudes were subjected to two separate GLM repeated measures analyses: first for the painful and second for non-painful conditions, which included three within-subject factors: Hand Position (2 levels: crossed and uncrossed), Cue (2 levels: congruent and incongruent) and Electrode Location (5 levels: Fz, FCz, Cz, CPz and Pz). The statistically significant main effects of Cue and Hand Position tested our hypothesis 4 and 5, respectively. In accordance to our a-priori hypotheses, F test was followed by planned comparisons tests when significant interactions of factors Cue and Hand Position were observed. First we investigated whether a cueing effect was present in both hand positions by performing planned comparisons for P3 peak amplitudes of congruently versus incongruently cued stimuli, separately for uncrossed and crossed hands conditions. Next, we performed planned comparisons for uncrossed hands versus crossed hands condition for P3 peak amplitudes elicited by incongruently cued stimuli (sixth hypothesis). Additionally, in order to investigate if crossing the hands reduced the magnitude of cueing effect, we performed a GLM for the difference of incongruent P3 and congruent P3 amplitudes (IC-CC difference) with Hand position (2 levels: crossed and uncrossed) as within-subject factor.

Additional post-hoc comparisons (p adjustment for multiple comparison: Bonferroni) were performed to investigate the presence of cueing effect on each of five midline electrodes. Additionally, in order to compare the magnitude of cueing effect between analysed electrodes, we performed a GLM for the difference of incongruent P3 and congruent P3 peak amplitudes (IC-CC difference) with Electrode Location (5 levels: Fz, FCz, Cz, CPz and Pz) as within-subject factor. Next, F test was followed by post-hoc comparisons (p value adjusted for multiple comparisons: Bonferroni).

The Cz latencies of the P3 components were analysed using a GLM with repeated measures with Cue (2 levels: congruent and incongruent) and Hand position (2 levels: crossed and uncrossed) as within-subject factors.

The Shapiro-Wilks test was used to assess the normality of distribution of investigated parameters. All parameters in our study were normally distributed. The level of significance was set at *p* < .05. Greenhouse–Geisser corrected *p*-values were reported where applicable. All the analyses were conducted using SPSS Version 22 (IBM Corporation, Armonk, NY, USA).

## Results

### 3.1. Behavioural results

**Fig 3:**
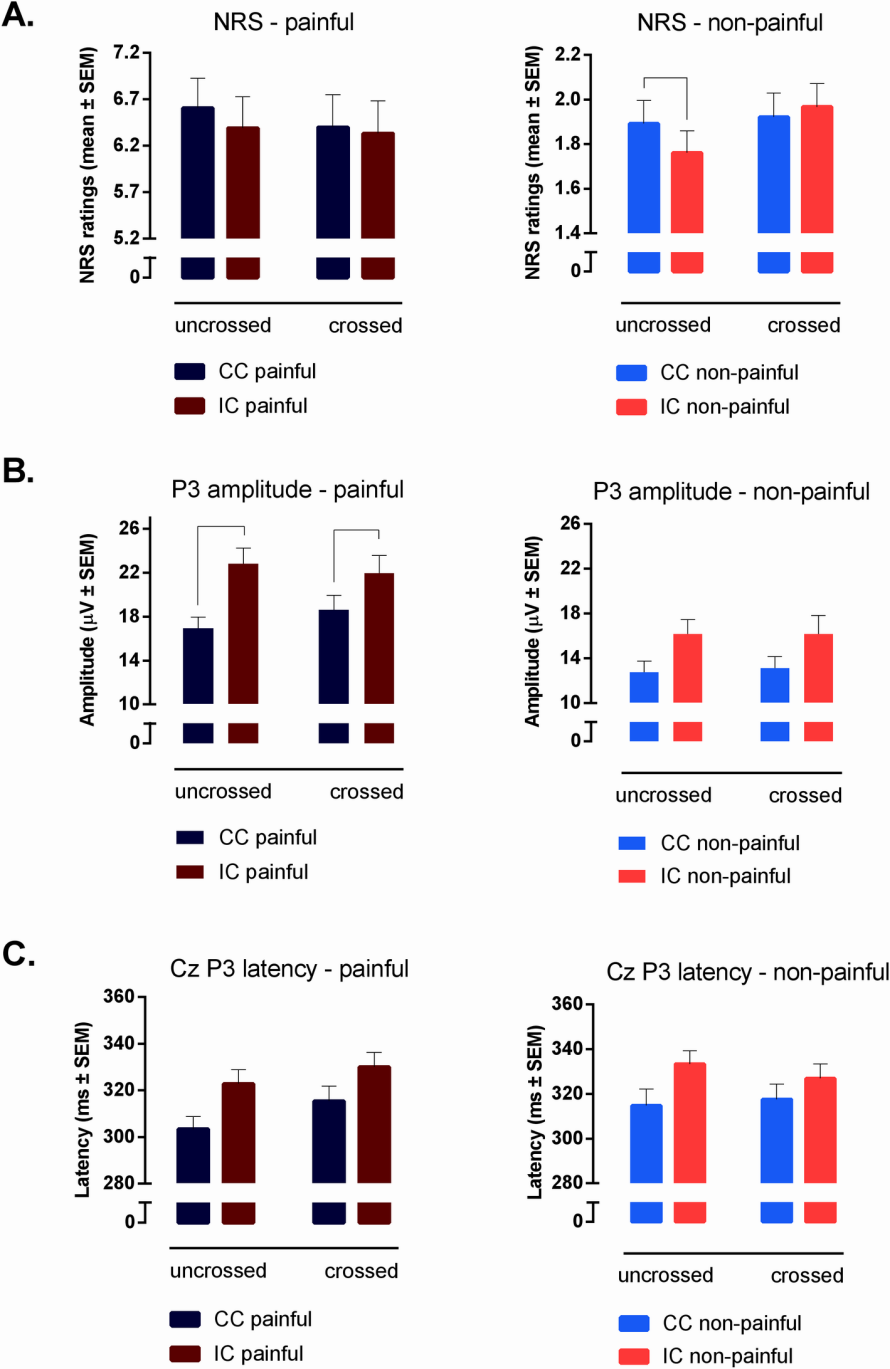
**Graphs of the behavioural (A) and neurophysiological results (B and C) of the study.** Behavioural results (NRS ratings) are presented in panel (A). P3 amplitudes and P3 latencies are presented in panels (B) and (C) respectively. Results for painful stimuli are presented on the left side of the figure and for non-painful stimuli on the right side.

The means of the NRS ratings of painful and non-painful stimuli in each of the four experimental conditions are presented in Fig 3A. The summary of the GLM with repeated measures for painful and non-painful NRS ratings is presented in Table 1.

**Table 1:** Summary of the GLM with repeated measures of the NRS ratings.

**NRS–Painful stimuli–the main effects and interaction effects (df)**	**F**	**p**	**ŋ^2^**
Cue (1,21)	**9.64**	**.005**	.05
Hand Position (1,21)	1.31	.27	.002
Cue × Hand Position (1,21)	2.71	.11	.01
**NRS–Non-painful stimuli–the main effects and interaction effects (df)**	**F**	**p**	**ŋ**^**2**^
Cue (1,21)	2.05	.17	.01
Hand Position (1,21)	2.93	.10	.01
Cue × Hand Position (1,21)	**8.85**	**.007**	**.05**

The behavioural results obtained for painful stimuli supported our first hypothesis. As was indicated by a statistically significant main effect of Cue, the NRS ratings were higher in the condition where the stimulus location was correctly signalled (congruent cueing) as compared to the condition where the stimulus location was incorrectly signalled (incongruent cueing). Moreover, the participants rated the stimuli on the NRS approximately equally in the crossed and uncrossed hands conditions indicated by the non-significant main effect of Hand Position (see Table 1). Thus, we did not observe crossed-hands analgesia as was suggested in the second hypothesis. Finally, a no significant interaction effect of Cue × Hand Position was observed, indicating that the cueing effect for painful stimuli was not significantly modulated by hand position which is not in line with our third hypothesis.

The behavioural results obtained for non-painful stimuli did not support our first (overall simple cueing effect) or second hypothesis (overall crossed-hands analgesia) as the main GLM revealed no statistically significant main effect for Cue or for Hand Position (see Table 1). At the same time, in contrast to our results on the painful stimuli, a significant Cue × Hand Position interaction was found, indicating that hand position may influence the magnitude of the cueing effect; this supports our third hypothesis. Given this effect, we performed separate planned comparisons for uncrossed and crossed hands condition to investigate whether a cueing effect was present in both conditions. The analysis revealed that the cueing effect was present only in the uncrossed hands condition—participants rated stimuli preceded by congruent cues as more intense than incongruent ones (*F*_(1,21)_ = 10.94, *p* = .003, ŋ^2^_p_ = .34). In the crossed hands condition we did not observe any effect of cueing (*F*_(1,21)_ = 1.03, *p* = .34, ŋ^2^_p_ = .05) (Fig 3A). Thus, crossing the hands over the body’s midline abolishes the cueing effect that can be observed in the uncrossed hand condition.

### 3.2. Electrophysiological results

#### P3 peak amplitude

**Fig 4:**
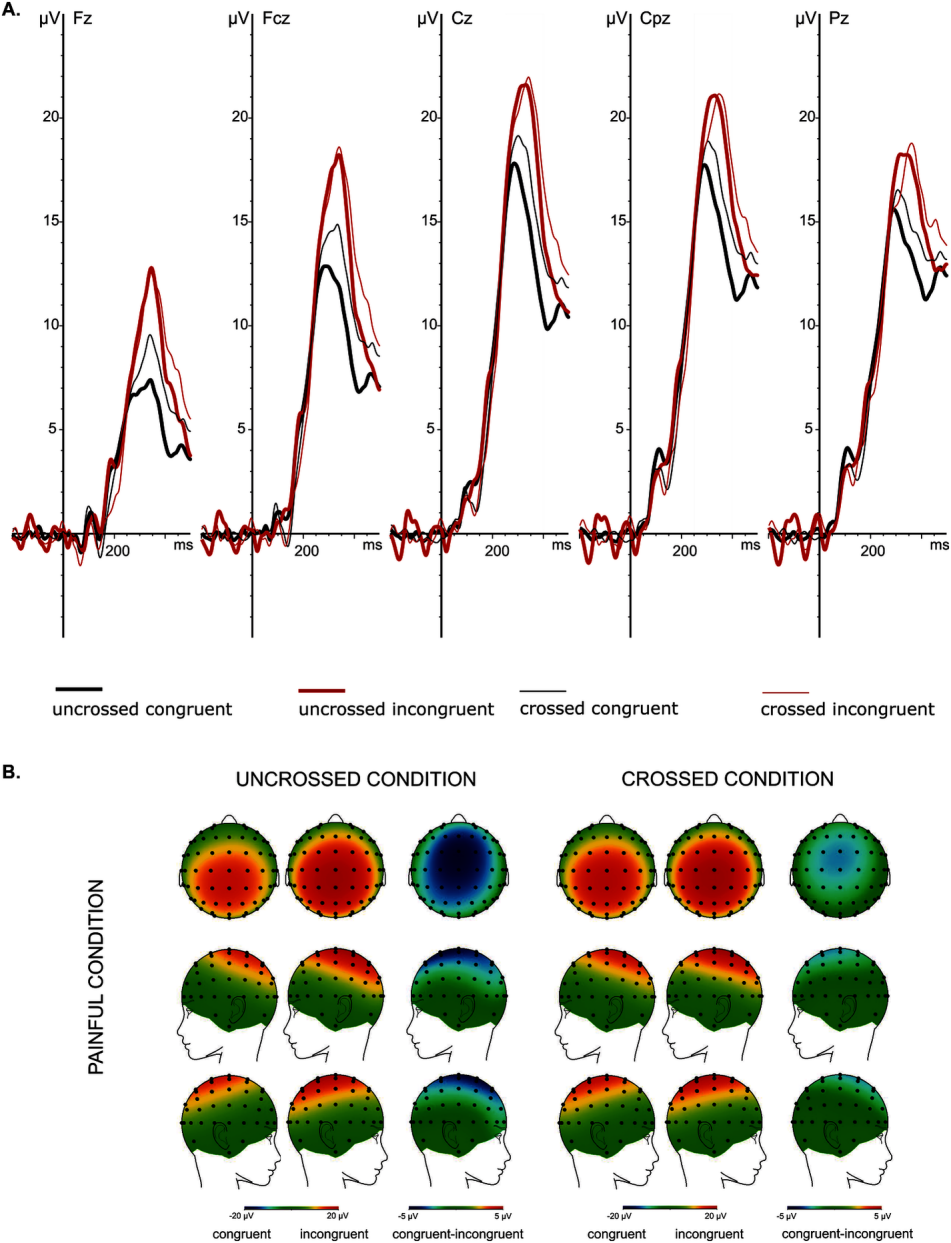
**Painful condition: the grand average ERPs from the midline electrodes (Fz, FCz, Cz, CPz and Pz) (A) and spline interpolated maps of potentials representing scalp top-views of the P3 (250–400 ms) as a function of Hand Position (uncrossed and crossed) and Cue (congruent and incongruent) (B).** Ad A: The highest P3 peak amplitudes were observed at Cz and CPz electrodes. Ad B: The uncrossed hands condition is on the left and the crossed hands condition is on the right. The colour scales—maximum and minimum—are coded in red and blue, respectively.

**Fig 5:**
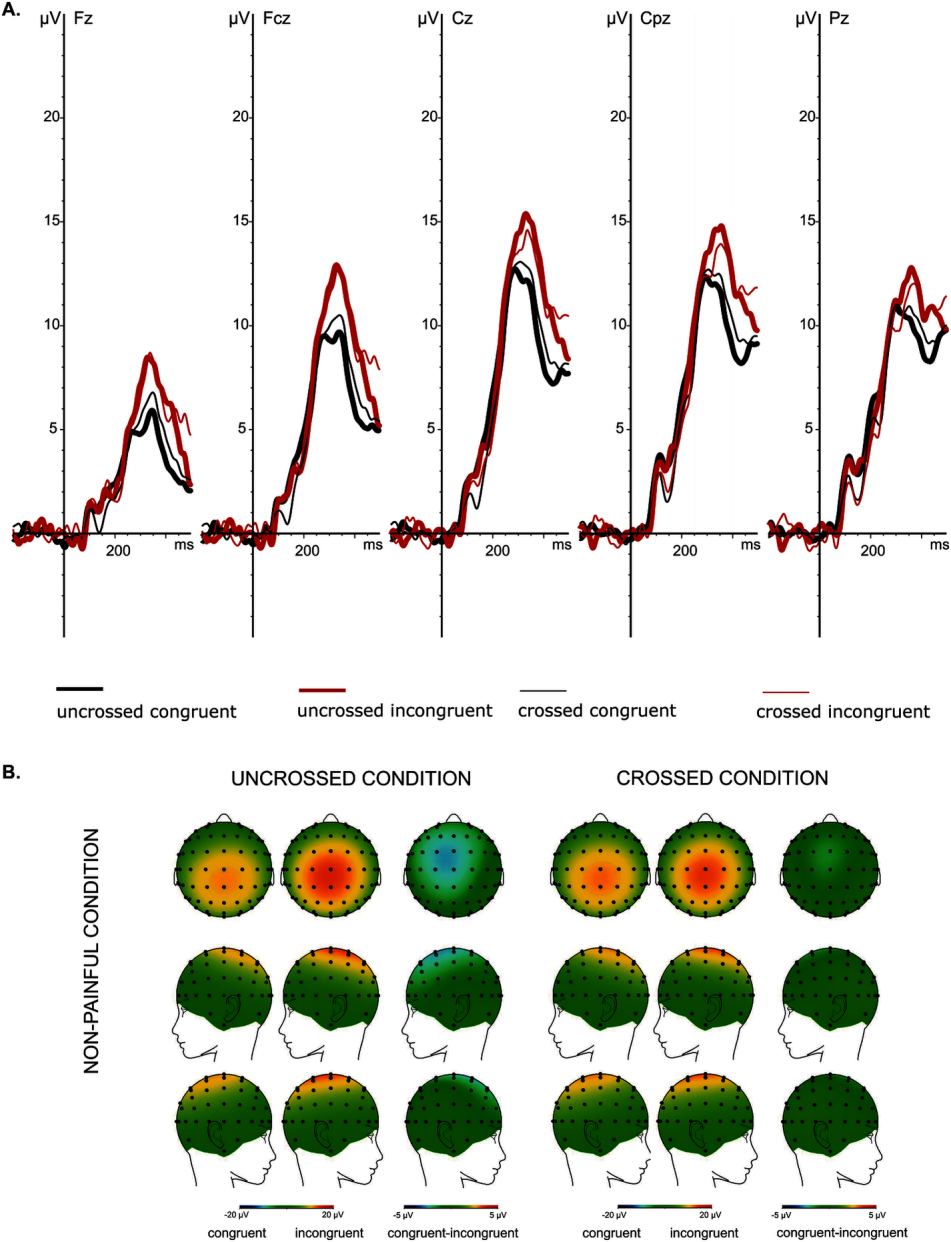
**Non-painful condition: the grand average ERPs from the midline electrodes (Fz, FCz, Cz, CPz and Pz) (A) and spline interpolated maps of potentials representing scalp top-views of the P3 (250–400 ms) as a function of Hand Position (uncrossed and crossed) and Cue (congruent and incongruent) (B).** Ad A: The highest P3 peak amplitudes were observed at Cz and CPz electrodes. Ad B: The uncrossed hands condition is on the left and the crossed hands condition is on the right. The colour scales—maximum and minimum—are coded in red and blue, respectively.

The summary of the GLM with repeated measures of the P3 peak amplitudes obtained for painful and non-painful stimuli is presented in Table 2. The P3 peak amplitudes recorded from the five midline electrodes in the four experimental conditions are presented in Fig 2B. The scalp topographies and the grand average ERPs recorded from the five midline electrodes in painful and non-painful conditions are presented in Figs 4 and 5, respectively.

**Table 2:** Summary of the GLM with repeated measures of the P3 amplitudes.

**P3 amplitude–Painful stimuli–the main effect and the interaction effects (df)**	**F**	**P**	**ŋ**^**2**^
Cue (1,21)	28.94	< .001	.08
Hand Position (1,21)	.34	.58	.001
Electrode Location (4,84)	80.53	< .001	.24
Cue × Hand Position (1,21)	8.62	.008	.01
Cue × Electrode Location (4,84)	6.09	.007	.002
Hand Position × Electrode Location (4,84)	.47	.62	.0001
Cue × Hand Position × Electrode Location (4,84)	1.20	.30	.0003
**P3 amplitude–Non-painful stimuli–the main effect and the interaction effects (df)**	F	P	ŋ^2^
Cue (1,21)	25.56	< .001	.06
Hand Position (1,21)	.08	.80	.0002
Electrode Location (4,84)	63.33	< .001	.16
Cue × Hand Position (1,21)	.17	.68	.0002
Cue × Electrode Location (4,84)	6.89	.003	.002
Hand Position × Electrode Location (4,84)	2.92	.07	.0008
Cue × Hand Position × Electrode Location (4,84)	.21	.73	.0001

The results of the GLM with repeated measures performed for P3 peak amplitude elicited by painful stimuli showed a significant main effect of Cue (fourth hypothesis). However, analogously to the NRS ratings to the painful stimuli, no significant main effect of Hand Position was observed (fifth hypothesis). Furthermore, the analysis revealed a significant Cue × Hand Position interaction. Planned comparison showed that the P3 peak amplitudes in response to painful stimuli preceded by incongruent cues were increased compared to those preceded by the congruent cues, in both the uncrossed (*F*_(1,22)_ = 32.01, *p* < .001, ŋ^2^_p_ = .60) and crossed hands conditions (*F*_(1,22)_ = 14.32, *p* = .001, ŋ^2^_p_ = .41) (Figs 3B and 4). Moreover, planned comparisons revealed that crossing the hands did not affect P3 peak amplitudes of the incorrectly cued painful stimuli (*F*_(1,22)_ = .85, *p* =. 37, ŋ^2^_p_ = .04) (sixth hypothesis). These analyses leave the found Cue × Hand Position interaction unexplained. A subsequent GLM for the amplitude difference of the incongruently and congruently cued stimuli did reveal a main effect of Hand position (*F*_(1,21)_ = 7.91, *p* = .01, ŋ^2^ = .04), indicating that the magnitude of the cueing effect of P3 amplitude of painful stimuli was more pronounced in uncrossed hands condition compared to crossed one (Fig 3B). This result indicates that crossing the hands reduces the magnitude of the cueing effect. A main GLM with repeated measures performed for P3 peak amplitude elicited by painful stimuli showed also a significant main effect of Electrode Location, with the largest values on the central sites (Cz and CPz) (Fig 4). Moreover a significant Cue × Electrode Location interaction was also observed (see Table 2). Further analysis showed that the cueing effect was present at five analysed vertex electrodes (post hoc ps < .001). Additional GLM analysis on IC-CC difference of P3 peak amplitudes elicited by painful stimuli showed that the magnitude of the cueing effect differed between analysed electrodes (*F*_(4,84)_ = 6,09, *p* = .01, ŋ^2^ = .03). Further analysis indicated that the effect of cueing was more pronounced at frontal-central sites (mean ± SD: Fz: 5.10 ± 4.21 μV, FCz: 5.46 ± 4.70 μV and Cz: 5.00 ± 4.48 μV) than at more posterior sites (mean ± SD: CPz: 4.17 ± 4.17 μV and Pz: 3.34 ± 3.74 μV).

For non-painful P3 peak amplitudes, the results of GLM with repeated measures showed a significant main effect of Cue, revealing a more pronounced P3 amplitude in response to stimuli following incongruent cues compared to congruent ones. Again, we obtained a non-significant main effect of Hand Position; thus, crossing the hands did not influence the P3 peak amplitude, which contradicts our fifth hypothesis. No Cue × Hand Position interaction effect was found, suggesting that the strength of the cueing effect is not different when measured in the uncrossed and crossed hands conditions (Fig 3B). Thus, the lack of statistical interaction runs contrary to our sixth hypothesis. A significant main effect of Electrode Location and significant Cue × Electrode Location interaction was found (see Table 2). Further post hoc analysis revealed that the cueing effect was present for each of five vertex electrodes (Fig 5). Similarly to the painful condition, we found that the P3 peak amplitudes elicited by incongruently cued non-painful stimuli were relatively larger in comparison to the P3 amplitudes of congruently cued stimuli. We also showed that the magnitude of the cueing effect (IC-CC difference) differed between the analysed electrodes (*F*_(4,84)_ = 6,89, *p* = .003, ŋ^2^ = .03). We found that the effect of cueing was less pronounced at Pz electrode (2.23 ± .63μV) compared to FCz, Cz and CPz electrodes (mean ± SD: 3.82 ± .70 μV, 3.56 ± .68 μV and 3.23 ± .71 μV, respectively) (Fig 5).

#### P3 latency

The summary of the GLM with repeated measures of the P3 latencies obtained separately for painful and non-painful stimuli is presented in Table 3.

**Table 3:** Summary of the GLM analysis of the P3 latencies.

**P3 latency–Painful stimuli–the main effect and the interaction effects (df)**	**F**	**P**	**ŋ**^**2**^
Cue (1,21)	9.02	.007	.07
Hand Position (1,21)	16.40	.001	.24
Cue × Hand Position (1,21)	.53	.47	.005
**P3 latency–Non-painful stimuli–the main effect and the interaction effects (df)**	**F**	**P**	**ŋ**^**2**^
Cue (1,21)	14.43	.001	.14
Hand Position (1,21)	.11	.75	.002
Cue × Hand Position (1,21)	1.75	.20	.02

The results revealed a significant main effect of Cue for both stimuli types, which is in line with our fourth hypothesis. This result suggests that P3 peaked later when elicited by stimuli following incongruent cues (mean ± SD: 326 ± 26 ms and 330 ± 25 ms for painful and non-painful stimuli respectively) compared to congruent ones (mean ± SD: 309 ± 26 ms and 316 ± 29 ms for painful and non-painful stimuli respectively) (Fig 3C). Interestingly, for painful stimuli, longer P3 latency was measured in the crossed (mean ± SD: 323 ± 27 ms) compared to the uncrossed hands condition (mean ± SD: 313 ± 24 ms), indicating that crossing the hands may have influenced P3 latency.

## Discussion

The present experiment used two manipulations to examine their effects on the perception and processing of painful and non-painful electrical stimuli. These were transient spatial attention manipulation, using congruent or incongruent cueing, and an alteration of the frame of reference by crossing the hands over the body midline. The perception of the stimuli was quantified with a behavioural measure, namely subjective scoring; the processing was quantified with an electrophysiological measure, namely the P3 of the evoked potential. Our study shows several main effects of cueing in different analyses, indicating that spatial attention influences both subjectively perceived intensity (NRS scores) and processing (P3 amplitude and P3 latency) of somatosensory stimuli. Except for the latency of the P3 following painful stimuli, we did not find other main effects of crossing the hands, demonstrating that crossing the hands over the body’s midline decreased neither subjective ratings nor the P3 peak amplitude of painful and non-painful stimuli. However, several Cue × Hand Position interaction effects were found in different analyses, indicating that crossing the hands modulates the effect of attention, though these modulating effects were only present for the non-painful NRS ratings and for P3 peak amplitudes following painful stimuli.

Our behavioural findings showed that NRS ratings of painful stimuli were modulated by the direction of spatial attention. The subjective NRS ratings were higher when attention was focused on the same side where the electrical stimulation occurred (congruent condition), compared to the ratings of the incongruent condition, which is consistent with our first hypothesis. These findings align with previous reports indicating that focusing attention on a nociceptive stimulus exaggerates pain [6,23]. Higher NRS scores of congruently cued painful stimuli could also be associated with the observation that when a region of the body’s space is cued by a stimulus in one modality, the processing of a stimulus from another modality appearing in that region is also facilitated [22,62]. For non-painful stimuli, the cueing effect failed to reach significance. However, for non-painful stimuli, we did observe a significant interaction effect (Cue x Hand Position) that will be discussed below.

Contrary to our second hypothesis, crossing the hands over the body midline did not significantly influence the NRS scores for either painful or for non-painful stimuli. The fact that we did not observe crossed-hands analgesia could be caused by a difference in the procedures of the present study compared to previous studies by others [15,17]. In the study by Gallace et al. [15], the side of the stimulus presentation was unknown to the participants; therefore, they did not know to which spatial location the stimuli would be delivered. This clearly contrasts with the present study, in which participants were cued with regard to the spatial location at which the stimulation would be delivered. In our study the side of the stimulus application was correctly cued in 80% of the trials; therefore the processing of painful and non-painful stimuli was facilitated (or inhibited with incongruently cued stimuli). The cueing effect might have overshadowed the hand position effect, possibly explaining the lack of crossed-hands analgesia. However, the study of Valentini et al. [16] proposed that vision is critical in eliciting crossed-hands analgesia thus questioning previous findings of Gallace et al. [15]. Since in the present study the participants could not see their hands (a table top blocked the view of their arms), the lack of visual information might explain why no crossed-hands analgesia was induced. Indeed, Valentini et al. [16] suggested that only the interaction between the crossed arms and an unobstructed view of the stimulated hand would be effective in reducing pain. As a result of these crucial differences, we might not have observed simple crossed-hands analgesia, but instead, observed the interaction between two different factors: the spatially guided allocation of attention with an internal (skin-based) frame of reference, and the externally, spatially guided allocation of attention.

The Cue × Hand Position interaction was seen for the non-painful stimuli, but not the painful ones, and therefore this finding is only partially in line with the third hypothesis. Observed differences in manipulation effects between the painful and non-painful conditions may be associated with the threatening character of painful stimuli, which inherently attract [63] and captures attention [64,65]. Thus, we assume that due to their saliency, painful stimuli are easy to localise even if the hands are kept in a crossed position. However, this lack of interaction effect in the painful condition cannot exclude the relevance of the external location, since the ERP results did reveal an interaction effect, discussed later.

In line with the third hypothesis, for non-painful stimuli, participants rated stimuli preceded by congruent cues as more intense in comparison to incongruent ones only in the uncrossed hands condition (Fig 3A). Thus, although no crossed-hands analgesia was observed, crossing the hands diminished the overshadowing cueing effects. It remains unclear if this effect relies on the same underlying mechanism as crossed-hands analgesia. However, a declined tactile localisation accuracy might explain why we did not observe an effect of cueing in the crossed hand condition [5,9,11,12,66]. The perception of non-painful stimuli (skin-based spatial code) appears to be automatically transferred to the external reference frame, which is known as tactile remapping [7,13,67,68]. Thus, skin-based codes and transformed external spatial codes are used in order to estimate the location of a non-painful stimulus [4,14,69]. In crossed hands condition, the processing of conflicting information from two different frames of reference is disturbed, which probably results in an abolishment of the cueing effect.

The effect of spatial attention allocation was also present for both the P3 peak amplitudes and P3 latencies of painful and non-painful stimuli. In line with the fourth hypothesis, the P3 components had higher amplitudes and prolonged latencies for incongruently cued trials as compared to congruently cued trials. For the P3 amplitude elicited by painful stimuli however, the magnitude of the cueing effect was reduced in the crossed hands condition as compared to the uncrossed hands condition, discussed later.

Our ERPs results suggest that the brain does respond differently to attended and unattended stimuli, reflecting an increased demand on attentional resources in the brain to locate a stimulus that is incongruently cued compared to a stimulus that is congruently cued [70]. Incongruent cues require re-allocation of attention, whereas attention is already focused at the correct position in the case of congruent cueing. The results are in line with the re-orienting response that has been reported in previous studies [33–35,38] and with the fact that noxious stimuli, such as pain, can attract our attention automatically, especially when they are unpredictable, intense, and/or novel [6,63]. Our ERPs results are also in line with previous findings showing that nociceptive and tactile stimuli capture participants’ attention, even when they are presented outside the focus of spatial attention [24,27,41–43].

Contrary to our fifth hypothesis, the ERP P3 peak amplitude elicited by both painful and non-painful stimuli was not decreased by crossing the hands. The lack of main effect of Hand Position in the present study is probably associated with the described differences between our study and that of Gallace et al. [15]. However, with respect to the painful stimuli we did find a main effect of crossing the hands in the P3 latency. Crossing the hands leads to more prolonged latencies, which suggests a delayed processing [39] of these stimuli due to a mismatch between internal and external references frames. Thus, we propose that spatial attention allocation has a cost in terms of processing resources–the brain has to realign skin-based coordinates to external spatial coordinates [9,15].

Contrary to our sixth hypothesis we did not observe that stimuli preceded by incongruent cues elicited higher P3 amplitude in uncrossed conditions compared to crossed conditions. However, the Cue × Hand Position interaction results obtained for the P3 peak amplitude following painful stimuli allowed us to indicate that crossing the hands reduces the magnitude of the cueing effect. The Cue × Hand Position interaction suggests that spatial attention is not guided by an internal (skin-based) reference frame alone (for tactile stimuli see [19,20]). In order to understand our results we may also exploit the knowledge from tactile [5,9,11,12,66,67] and pain [11] information processing studies in which the temporal order judgment (TOJ) task was used. In those studies an increase in localization errors was observed in the crossed hands condition (a crossed-hands deficit). Thus the reduction of the magnitude of the cueing effect in the crossed hands condition may reflect a conflict between internal and external left–right coordinates that decreases the ability to localize stimuli. The processing of the stimuli location is not disturbed when the hands are uncrossed, since in this situation the internal and external reference frames provide congruent information.

For P3 peak amplitude elicited by non-painful stimuli we have observed a main effect of Cue and have not observed a main effect of crossing the hands nor an interaction effect of Cue and Hand Position. This result indicates that a comparable magnitude of the cueing effect was observed in both hand conditions. In a study of Heed and Röder [20], where also tactile stimuli were used, a difference in the 190–300 msec window of the ERPs between attended and unattended stimuli was observed for the crossed but not the uncrossed condition. However, the manipulation of attention, sustained in their study and transient in our experiment, was what may have led to differences between the results of the mentioned study and ours. Moreover, the ERP in the Heed and Röder [20] study, to which we referred, is not the P3, so a direct comparison cannot be made.

In the present study we investigated the P3 component recorded from five vertex electrodes. The results suggest that our P3 component elicited by electric stimuli seems most likely the equivalent of the P3a component due to the fact that the amplitudes of the P3 were greater at the Cz than at the Pz electrode. Analogously, to our results, in the study of Van der Lubbe et al. [44], where transient attention using a Posner task was studied, the amplitude of the P260 component was also maximal at Cz and was larger for unattended compared to attended stimuli. The authors assumed that the P260 is an equivalent of the P3a component. Moreover, the P260 component was assumed to be the nociceptive correlate of the P3a component (for discussion see [40–42]), Another study confirms this, showing that this component was enhanced by rarely presented deviant laser stimuli [27]. Moreover, in the present study we also observed that the cueing effect was more pronounced at fronto-central sides.

Some limitations of the current study should be discussed. The behavioural and ERP results show different effects of the experimental manipulations. In the crossed hands condition for non-painful stimuli, the NRS scores show no cueing effect, whereas for the painful condition the P3 amplitudes show a decrease in magnitude of this cueing effect. This discrepancy of behavioural and EEG data is common as was shown in previous studies [71,72]. One of the explanations for such a pattern of results is that behavioural data are less sensitive because they are the end product of all preceding cumulative cognitive operations and thus more vulnerable to noise and variability. Interestingly, in an additional analysis, we only found a correlation for the least demanding cognitive task, i.e. a correlation between the NRS and P3 amplitude for congruent cued painful stimuli in the uncrossed hands position (S2 Fig). One drawback is that the electrical painful and non-painful stimuli measurements were based on participants’ ratings on the NRS (subjective measurements), the veracity of which may be questioned [44]. However, objective measurements obtained from the ERP components provide a useful method for investigating the physiological effects of modulated pain perception [28,73–75]. Another limitation is that in our study we collected painful ratings and recorded EEG activity at the same time, so it is possible that our results reflect the effect of cognitive enhancement of pain-related brain activity. An alternative approach would be to allow participants to assess pain at the end of each block, long after the stimuli were given, but this would likely produce unreliable NRS scores due to the long delay between the painful stimulus and its rating. Furthermore, such a design might measure participants’ beliefs about pain–this could introduce differences in stimulus intensities in the congruent and incongruent cue conditions [76]. Valentini et al. [16] used another approach where two separate experiment sessions were used for behavioural and psychophysiological measurements. In one session participants were asked to evaluate their pain sensation according to the experimental conditions and in the following sessions only ERPs were collected during the very same conditions. Such a design would have prevented pain-related brain activity’s being affected by cognitive enhancement. The next limitation is that in the present study the painful and non-painful stimuli were evaluated for the non-dominant (left) hand and during the experiment such individually determined stimuli were also used for the dominant one. The threshold of pain perception may vary considerably for the dominant and non-dominant hand; however, reports concerning this topic are conflicting [77,78]. Finally, it should be emphasised that in conducting research on pain and attention, one needs to be aware that any interruptive effects of pain could be attributed to affective processing (e.g. fear or anxiety) taking place during the anticipation of pain. There is evidence that fear of pain is associated with stronger pain-related brain activity [79]. As a result, the enhanced P3 amplitudes may merely reflect an increased fear of pain.

In conclusion, both the behavioural and ERP data indicated that the manipulation of transient spatial attention was effective and that hand position may modulate this cueing effect. Our results may have an implication for the development of therapies for chronic pain, which could focus on the use of attentional training as a pain management strategy. Such attentional training may facilitate the ability to focus attention away from the painful body part and this may lead to pain reduction.

## Supporting information

S1 Fig Graphical representation of the hypotheses for both painful and non-painful stimuli. Panels on the left (A-C) depict the hypotheses with respect to the NRS ratings. Panels on the right (D-F) depict the hypotheses with respect to the ERP P3 component. Note: Dark grey colours represent expected increments (a.u.) whereas light grey colours represent expected decrements of scores. However, the grey colours do not quantify the effect and have only illustrative meaning. The horizontal and vertical rectangles (Match and Mismatch) represent relation between both frames of reference.
(TIF)

S2 Fig Correlation of individual NRS scores with P3 peak amplitude. (TIF)
