# Supplementary figures and images for: Influence of transient spatial attention on the P3 component and perception of painful and non-painful electric stimuli in crossed and uncrossed hands positions
Source: PLoS One. 2017 Sep 5;12(9):e0182616. doi: 10.1371/journal.pone.0182616 (PMC5584947; doi:10.1371/journal.pone.0182616)

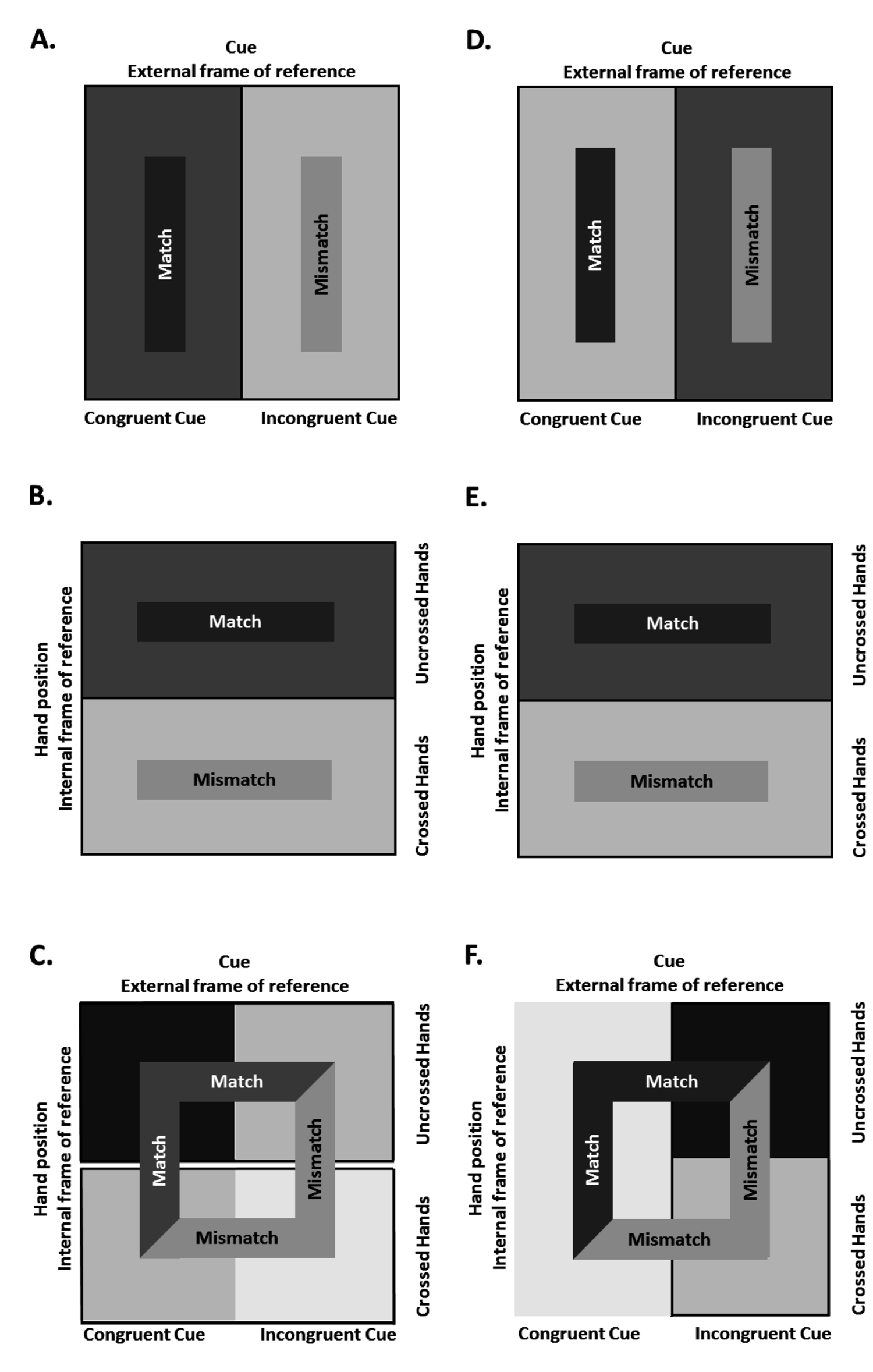

Supplement: S1 Fig — Panels on the left (A-C) depict the hypotheses with respect to the NRS ratings. Panels on the right (D-F) depict the hypotheses with respect to the ERP P3 component. Note: Dark grey colours represent expected increments (a.u.) whereas light grey colours represent expected decrements of scores. However, the grey colours do not quantify the effect and have only illustrative meaning. The horizontal and vertical rectangles (Match and Mismatch) represent relation between both frames of reference. (TIF) [file pone.0182616.s001.tif]
